# The IL-17A/TNFSF10 axis promotes vulvovaginal candidiasis pathogenesis by inducing neutrophil necroptosis

**DOI:** 10.3389/fcimb.2026.1910964

**Published:** 2026-09-24

**Authors:** Mingkun SHAO, Wanqin HU, Hongxiao FENG, Sainan LI, Wenjin QI, Yang LIU

**Affiliations:** 1 Department of Obstetrics and Gynecology, The Second Affiliated Hospital of Kunming Medical University, Yunnan, China; 2 Department of Obstetrics and Gynecology, The First Affiliated Hospital of Kunming Medical University, Yunnan, China

**Keywords:** interleukin-17A, necroptosis, neutrophils, TNFSF10, vulvovaginal candidiasis

## Abstract

**Introduction:**

Vulvovaginal candidiasis (VVC) is a common infection of the female reproductive tract and is closely associated with dysregulated mucosal immunity. This study investigated how IL-17A/TNFSF10 signaling regulates neutrophil death and VVC progression.

**Methods:**

Clinical specimens, RNA sequencing, cell-based assays, and mouse models were used.

**Results:**

IL-17A enhanced neutrophil extracellular trap formation and increased TNFSF10/TNFRSF10 expression, together with increased phosphorylation of RIP3 and MLKL. *In vivo*, IL-17A aggravated mucosal injury and neutrophil infiltration, whereas TNFSF10 manipulation altered neutrophil accumulation, inflammatory responses, and epithelial barrier function.

**Discussion:**

These findings suggest that IL-17A/TNFSF10 signaling contributes to neutrophil death, mucosal inflammation, and VVC progression. Targeting this pathway may represent a potential therapeutic strategy for VVC.

## 1 Introduction

Vulvovaginal candidiasis (VVC) is an inflammatory infection of the vulva and vagina caused by *Candida* species and occurs most frequently in women of reproductive age. Typical manifestations include vulvovaginal itching, burning, irritation, soreness, and abnormal discharge. Recurrent vulvovaginal candidiasis (RVVC), commonly defined as four or more clinically confirmed symptomatic episodes within one year, imposes a substantial burden because of repeated symptoms and frequent relapse (Ilkit and Guzel, 2011). The pathogenesis of VVC reflects disrupted homeostasis between the host and the vaginal microbiota. *Candida albicans* is an opportunistic commensal of the vaginal tract that can overgrow and trigger inflammation when the local microenvironment and mucosal immunity are disturbed (Rautemaa-Richardson et al., 2026). Dysregulation of both innate and adaptive immunity contributes to recurrence and progression to RVVC (MacAlpine and Lionakis, 2024).

IL-17A was selected as the upstream focus of this study because it is a major mediator of mucosal antifungal immunity and regulates neutrophil recruitment and activation during *Candida* infection (Mara et al., 2022). Previous VVC studies, including our murine work, showed that IL-17 signaling shapes vaginal neutrophil responses (Mara et al., 2022; Shao et al., 2023). Nevertheless, neutrophils may become pathogenic in the dysregulated vaginal microenvironment: excessive recruitment amplifies inflammation (Mara et al., 2022), while impaired antifungal activity limits effective clearance of *C. albicans* (Richardson et al., 2018). We therefore examined whether IL-17A-driven neutrophil responses converge on a cell-death pathway that contributes to local tissue injury.

Apoptosis, necroptosis, and neutrophil extracellular trap (NET) formation are major neutrophil death programs that differentially influence inflammation and tissue damage (Tu et al., 2024). TNFSF10 (TRAIL/CD253), a member of the tumor necrosis factor superfamily, can induce apoptosis (Zhang et al., 2026) and, under specific pathological conditions, engage RIPK1/RIP3/MLKL-associated necroptosis signaling (Wicki et al., 2016; Wang et al., 2024). NETs also have context-dependent effects in VVC: they can trap *C. albicans* (Johnson et al., 2016), whereas excessive or dysregulated NET formation may sustain inflammation and aggravate mucosal injury (Roselletti et al., 2023).

To investigate the IL-17A/TNFSF10 axis in VVC, we combined transcriptomic screening, molecular validation, *in vitro* assays, an *in vivo* mouse model, and an additional TNFSF10-knockdown rescue experiment. We evaluated whether IL-17A alters TNFSF10/TNFRSF10 signaling and neutrophil necroptosis, and whether TNFSF10 silencing reverses IL-17A-induced RIP3/MLKL activation, inflammatory mediator production, and LDH release.

## 2 Materials and methods

### 2.1 Collection of clinical samples and isolation and culture of neutrophils

The clinical study was approved by the Ethics Committee of the Second Affiliated Hospital of Kunming Medical University (approval no. PJ-KY-2026-17), and written informed consent was obtained from all participants. Vaginal lavage samples were collected from 12 patients with VVC caused by *C. albicans* and 12 healthy volunteers between January 2024 and January 2025. VVC was defined by compatible clinical symptoms (including vulvovaginal itching, burning, irritation, abnormal discharge, or discomfort) together with laboratory confirmation of *C. albicans* by microscopic examination and/or fungal culture. Healthy controls had no symptoms of vulvovaginal infection, negative fungal examination results, and no history of recurrent VVC. After centrifugation, cell pellets were processed, filtered, and prepared as single-cell suspensions. Neutrophils were isolated by density-gradient centrifugation, washed, and cultured in complete RPMI-1640 medium. Cells were assigned to four groups: Control, VVC, Control + IL-17A (100 ng/mL for 4 h), and VVC + IL-17A (100 ng/mL for 4 h). Samples were then used for transcriptomic sequencing, Western blotting, and immunofluorescence analyses.

### 2.2 Immunofluorescence detection of neutrophil extracellular traps (NETs)

Following the group-specific treatments described in Section 2.1, isolated neutrophils were incubated with or without IL-17A (100 ng/mL) for 4 h; no separate chemical NET inducer was used. Cells were then fixed with paraformaldehyde, washed three times with PBS, and incubated with SYTOX Green (Invitrogen, Cat. No. 2720299; 1:500) at 37 °C for 15 min in the dark to label extracellular DNA. After three 5-min PBS washes, nuclei were counterstained with Hoechst solution (Invitrogen, Cat. No. 62249; 1:10,000) for 10 min at room temperature in the dark, followed by three additional PBS washes. Images were acquired from six randomly selected fields under identical fluorescence-microscopy settings. NET formation was quantified as the percentage of NET-positive cells.

### 2.3 Transcriptomic analysis

To investigate how IL-17A regulates neutrophils in VVC, three vaginal lavage samples from each clinical group were used for transcriptomic analysis. Neutrophils from healthy donors were additionally treated with IL-17A. RNA sequencing was performed in three groups (n = 3 per group): Control (Group A), Control + IL-17A (Group B), and VVC (Group C). Total RNA extraction, library construction, sequencing, reference-genome alignment, and differential-expression analysis were performed to identify candidate genes and pathways.

Differentially expressed genes (DEGs) in Group B versus Group A and Group C versus Group A were identified using |log2 fold change| ≥ 0.5 and P < 0.05. Volcano plots were generated to visualize the direction and magnitude of differential expression.

Hierarchical clustering was performed using log2-transformed DEG expression values. The 20 most significant Gene Ontology terms and Kyoto Encyclopedia of Genes and Genomes pathways were visualized using bubble plots. Shared DEGs between the two comparisons were identified by Venn analysis and evaluated using protein-protein interaction networks and Cytoscape. Genes with degree > 10 were subjected to functional-enrichment analysis.

### 2.4 Detection of neutrophils in vaginal lavage fluid from clinical samples by qRT-PCR

Total RNA was extracted using TRIzol reagent (Ambion, Cat. No. 15596-018CN), and RNA concentration and purity were measured with a NanoDrop ND-1000 spectrophotometer. First-strand cDNA was synthesized using a commercial kit (Servicebio, Cat. No. G3336-50) and diluted fivefold. qRT-PCR was performed using SYBR Green qPCR Master Mix (Servicebio, Cat. No. G3328-01) and gene-specific primers. Relative transcript abundance was calculated using the 2−ΔΔCt method. Primers are displayed in **Table 1**.

**Table 1:** Primer sequences for human neutrophil qRT-PCR.

Primer	Sequence (5′→3′)
*GAPDH F*	CCCATCACCATCTTCCAGG
*GAPDH R*	CATCACGCCACAGTTTCCC
*TNFRSF10B F*	TGCTCTAACTGCACTGACGG
*TNFRSF10B R*	GGTCGTCAGCTGAGTCGTTT
*TNFSF10 F*	TTGGGACCCCAATGACGAAG
*TNFSF10 R*	TGGTCCCAGTTATGTGAGCTG
*IRF7 F*	GACGAAACTTCCCGTCCCG
*IRF7 R*	GCTGCCTCGGTATGGATCTC
*IFNA F*	TGAGACCCACAGCCTGGATA
*IFNA R*	CTGGAGCCTTCTGGAACTGG
*IFNB F*	CGAATTCTAGAGTCCCATACCCA
*IFNB R*	GACAACACGAACAGTGTCGC
*CXCL11 F*	CTGCCCAAAGGAGTCCAACA
*CXCL11 R*	TGTCTCCACCGTAACCACAG

### 2.5 Western blot

Cells were lysed in ice-cold RIPA buffer containing protease and phosphatase inhibitors. After centrifugation (16,000 × g, 4 °C, 10 min), protein concentrations were determined using the BCA assay. Equal amounts of protein were denatured, separated by 10% SDS-PAGE, and transferred to PVDF membranes (300 mA, 4 °C, 1 h). After blocking with 5% BSA, membranes were incubated overnight at 4 °C with primary antibodies against TNFSF10, TNFRSF10, RIP3, p-RIP3, MLKL, and p-MLKL (1:1,000), or β-actin (1:25,000). After TBST washing, HRP-conjugated secondary antibodies were applied for 1 h. Bands were visualized by enhanced chemiluminescence and quantified using ImageJ, with β-actin as the loading control.

### 2.6 Animal models

Thirty-six specific-pathogen-free female C57BL/6 mice (6–8 weeks old; 18–22 g) were obtained from Beijing SPF Biotechnology Co., Ltd. (production license: SCXK [Jing] 2024-0001; facility use license: SYXK [Dian] K2022-0007). Mice were housed under SPF conditions with free access to food and water and randomly assigned to six groups (n = 6 per group): (1) Control, without intervention; (2) VVC, receiving subcutaneous estradiol (1 mg/mL, 0.1 mL/mouse) on day 1 and every other day for three doses, followed by estrous-phase confirmation and intravaginal *C. albicans* inoculation; (3) VVC + OE-TNFSF10, receiving an intravaginal TNFSF10-overexpression vector (1 × 10¹² vector genomes/mL; 1 × 10¹^0^ vector genomes/mouse) before VVC induction; (4) VVC + sh-TNFSF10, receiving intravaginal sh-TNFSF10 at the same vector concentration and dose before VVC induction; (5) VVC + IL-17A, receiving recombinant IL-17A intraperitoneally (50 U/mouse) every 3 days for three doses before VVC induction; and (6) VVC + IL-17A nAb, receiving an IL-17A-neutralizing antibody intraperitoneally (50 U/mouse) every 3 days for three doses before VVC induction. Vaginal lavage fluid was examined for hyphal formation before sacrifice and tissue collection.

Vaginal lavage fluid, vaginal tissue, and blood neutrophils were collected for downstream analyses. All animal procedures followed the 3R principles and were approved by the Institutional Animal Care and Use Committee of Kunming Medical University (approval no. kmmu2020178).

### 2.7 Hematoxylin and eosin staining

Vaginal tissues were fixed in 4% paraformaldehyde, embedded in paraffin, sectioned at 3 μm, stained with hematoxylin and eosin, mounted, and scanned for histopathological evaluation.

### 2.8 Immunohistochemistry

Sections were baked, deparaffinized, and rehydrated. Antigen retrieval was performed in citrate buffer under high pressure, followed by blocking with 3% H_2_O_2_ and 5% BSA. Sections were incubated with an anti-ZO-1 antibody (Bioss, bs-34023R; 1:200), followed by signal enhancer and secondary antibody. After DAB development and hematoxylin counterstaining, slides were dehydrated, mounted, and scanned.

### 2.9 Flow-cytometric quantification of neutrophils

Cells were collected by centrifugation at 300 × g for 5 min, washed with PBS, and resuspended in 50 μL of 1× PBS. Samples were incubated with 5 μL of anti-mouse Ly6G-FITC antibody (Elabscience, E-AB-F1120D) for 30 min at room temperature in the dark. After addition of 400 μL of 1× binding buffer, suspensions were filtered through nylon mesh and analyzed on a BD FACSCalibur flow cytometer (≥10,000 events per sample). Ly6G^+^ neutrophils were quantified using FlowJo v10.8.1.

### 2.10 Isolation and co-culture of mouse neutrophils

Anticoagulated mouse whole blood was diluted 1:1 with PBS. Neutrophils were purified by density-gradient separation using the Solarbio P9201 isolation kit and cultured in T25 flasks at 37 °C in 5% CO_2_. Five experimental groups were established as follows:

(1) NEU, untreated neutrophils cultured for 4 h; (2) NEU + *C. albicans*, neutrophils co-cultured with *C. albicans* for 4 h; (3) NEU-si-NC + *C. albicans*, negative-control siRNA-transfected neutrophils co-cultured with *C. albicans* for 4 h; (4) NEU-si-TNFSF10 + *C. albicans*, TNFSF10-silenced neutrophils co-cultured with *C. albicans* for 4 h; and (5) NEU + *C. albicans* + IL-17A, neutrophils co-cultured with *C. albicans* and treated with IL-17A (50 ng/mL) for 4 h.

After treatment, cells were collected for immunofluorescence staining, flow cytometry, Western blotting, and qRT-PCR.

### 2.11 Immunofluorescence detection of TNFSF10 and TNFRSF10

Samples were fixed overnight in 4% paraformaldehyde at room temperature. After PBS washing, antigen retrieval was performed with pepsin at 37 °C for 30 min. Samples were permeabilized, blocked with 5% BSA, and incubated overnight at 4 °C with primary antibodies against TNFSF10 and TNFRSF10 (1:50 each), followed by rewarming at 37 °C for 30 min. Alexa Fluor 488- and 555-conjugated secondary antibodies were applied at 37 °C for 30 min. Nuclei were counterstained with DAPI, and five to six randomly selected fields were imaged by fluorescence microscopy.

### 2.12 Neutrophil migration assay

Purified neutrophils were resuspended in serum-free medium at 2.5 × 10^4^ cells/mL. A 200-μL cell suspension was placed in the upper chamber of each Transwell insert, and 500 μL of medium containing 10% FBS was added to the lower chamber. After incubation for 24 h at 37 °C in 5% CO_2_, cells that migrated to the lower chamber were fixed, stained with crystal violet, and counted in five randomly selected microscopic fields.

### 2.13 qRT-PCR analysis of mouse neutrophils

Total RNA was reverse-transcribed using a Yungen reverse-transcription kit. qPCR was performed with Saiwei SYBR Green reagent, and relative transcript abundance was calculated using the 2−ΔΔCt method. Primers are displayed in **Table 2**.

**Table 2:** Primer sequences for mouse neutrophil qRT-PCR.

Primer	Sequence
*Tnfsf10 F*	GCCAGCTCTGCTGTTTTGAG
*Tnfsf10 R*	CACCTGGTGGCACTAATGGT
*Tnfrsf10 F*	CACGGAACCTGGCAAGACT
*Tnfrsf10 R*	TGTGACCAGTGTTTCGGCTT
*Ripk3 F*	GCCTTCCTCTCAGTCCACAC
*Ripk3 R*	CTCACCAGAGGAACCGCATA
*Mlkl F*	GGCATTCGAGGAAAGGCAAAA
*Mlkl R*	ACACGGTTTCCTAGACGCTG
*Gapdh F*	TGTGTCCGTCGTGGATCTGA
*Gapdh R*	GAGTTGCTGTTGAAGTCGCA

### 2.14 *In vitro* rescue experiment, ELISA, LDH release assay, and Western blotting

To determine whether TNFSF10 mediates IL-17A-induced neutrophil necroptosis during fungal exposure, mouse neutrophils were assigned to four rescue groups: Control, *C. albicans*, *C. albicans* + IL-17A, and *C. albicans* + IL-17A + si-TNFSF10. After the indicated treatments, culture supernatants and cellular proteins were collected.

IL-1β and MPO concentrations in culture supernatants were quantified by ELISA, and LDH release was measured as an indicator of membrane injury and cell death according to the manufacturers’ instructions. TNFSF10, RIP3, p-RIP3, MLKL, p-MLKL, and β-actin protein expression was examined by Western blotting as described in Section 2.5, with β-actin used as the loading control.

### 2.15 Statistical analysis

Statistical analyses were performed using GraphPad Prism 9.0. Data are presented as the mean ± standard error of the mean (SEM). Two groups were compared using an unpaired Student’s t-test, and multiple groups were compared using one-way analysis of variance. *p* < 0.05 was considered statistically significant.

## 3 Results

### 3.1 IL-17A promotes NET formation in neutrophils isolated from human vaginal lavage fluid

Previous murine studies showed that IL-17A promotes vaginal neutrophil recruitment and modulates neutrophil death during VVC (Shao et al., 2023). Here, neutrophils isolated from healthy donors and patients with VVC were incubated with or without IL-17A (**Figure 1A**), and NET formation was evaluated by immunofluorescence.

**Figure 1:**
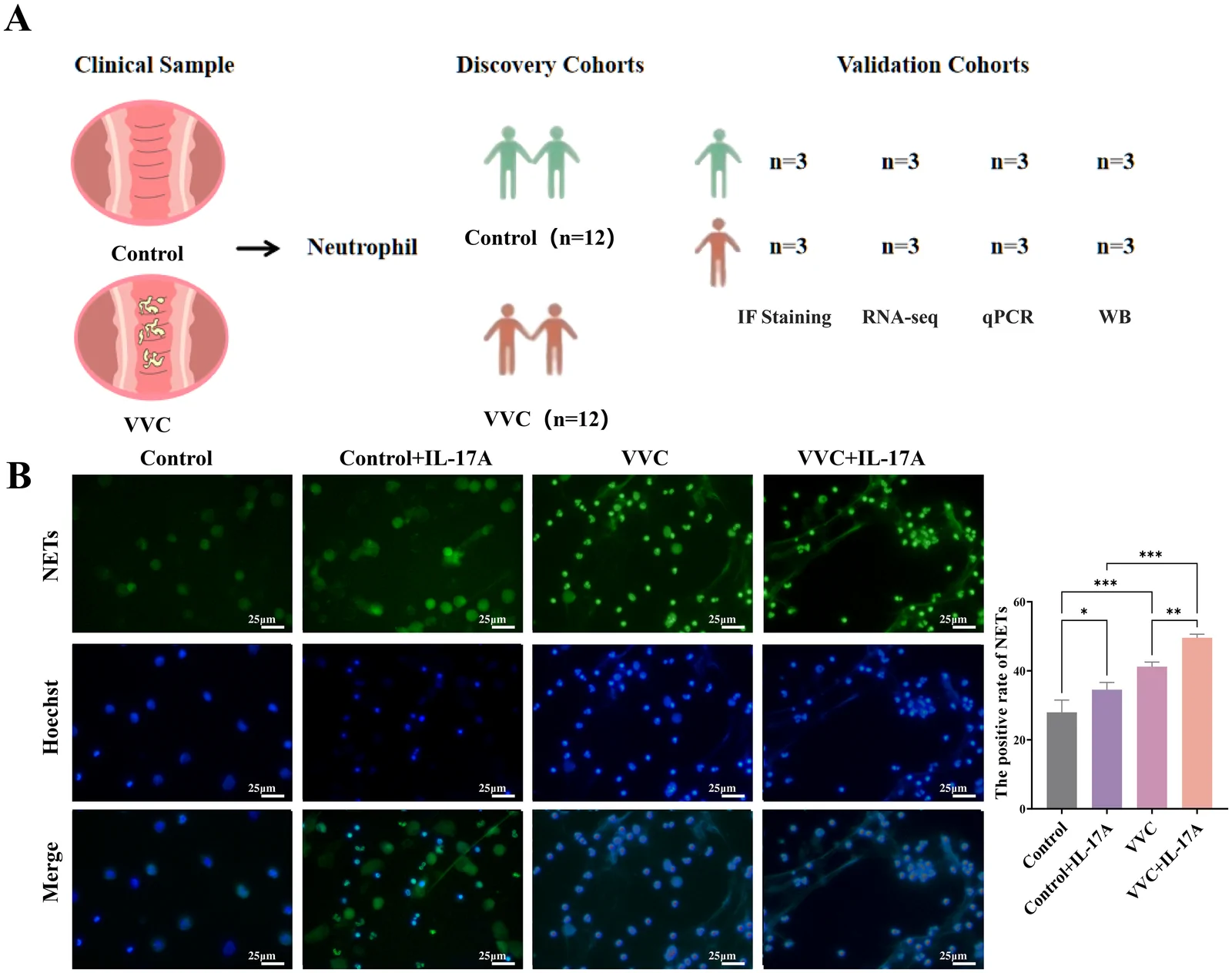
Effect of IL-17A on NET formation in neutrophils isolated from vaginal lavage fluid of healthy controls and patients with VVC. **(A)** Study design and sample allocation. **(B)** Enlarged, high-resolution representative immunofluorescence images and quantitative analysis of NET-positive cells. Green, extracellular DNA/NET signal; blue, nuclei. Scale bars = 50 mm. *p < 0.05; **p < 0.01; ***p < 0.001; ****p < 0.0001.

Basal NET formation was higher in neutrophils from patients with VVC than in those from healthy controls. IL-17A stimulation further increased NET-associated fluorescence and the percentage of NET-positive cells in both donor groups (**Figure 1B**). These findings indicate that IL-17A promotes NET formation in vaginal neutrophils and may contribute to local inflammatory responses in VVC.

### 3.2 Transcriptomic profiling identifies TNFSF10 as a candidate regulatory molecule

After confirming the effect of IL-17A on NET formation, we investigated the molecular response of vaginal neutrophils. RNA sequencing was performed using neutrophils from healthy controls (Group A), IL-17A-treated healthy controls (Group B), and patients with VVC (Group C). Group B versus Group A yielded 89 DEGs (58 upregulated and 31 downregulated), whereas Group C versus Group A yielded 1,574 DEGs (408 upregulated and 1,166 downregulated; **Figure 2A**). Venn analysis identified 55 shared DEGs (45 upregulated and 10 downregulated; **Figure 2B**). Network and enrichment analyses linked these genes to cytokine signaling, innate immune pathways, and necroptosis-associated pathways (**Figures 2C–G**). *TNFSF10*, *IRF7*, and *CXCL11* were represented in multiple enriched pathways. qRT-PCR confirmed higher transcript levels of these genes in Group C than in Group A, with further increases after IL-17A treatment (**Figure 2H**).

**Figure 2:**
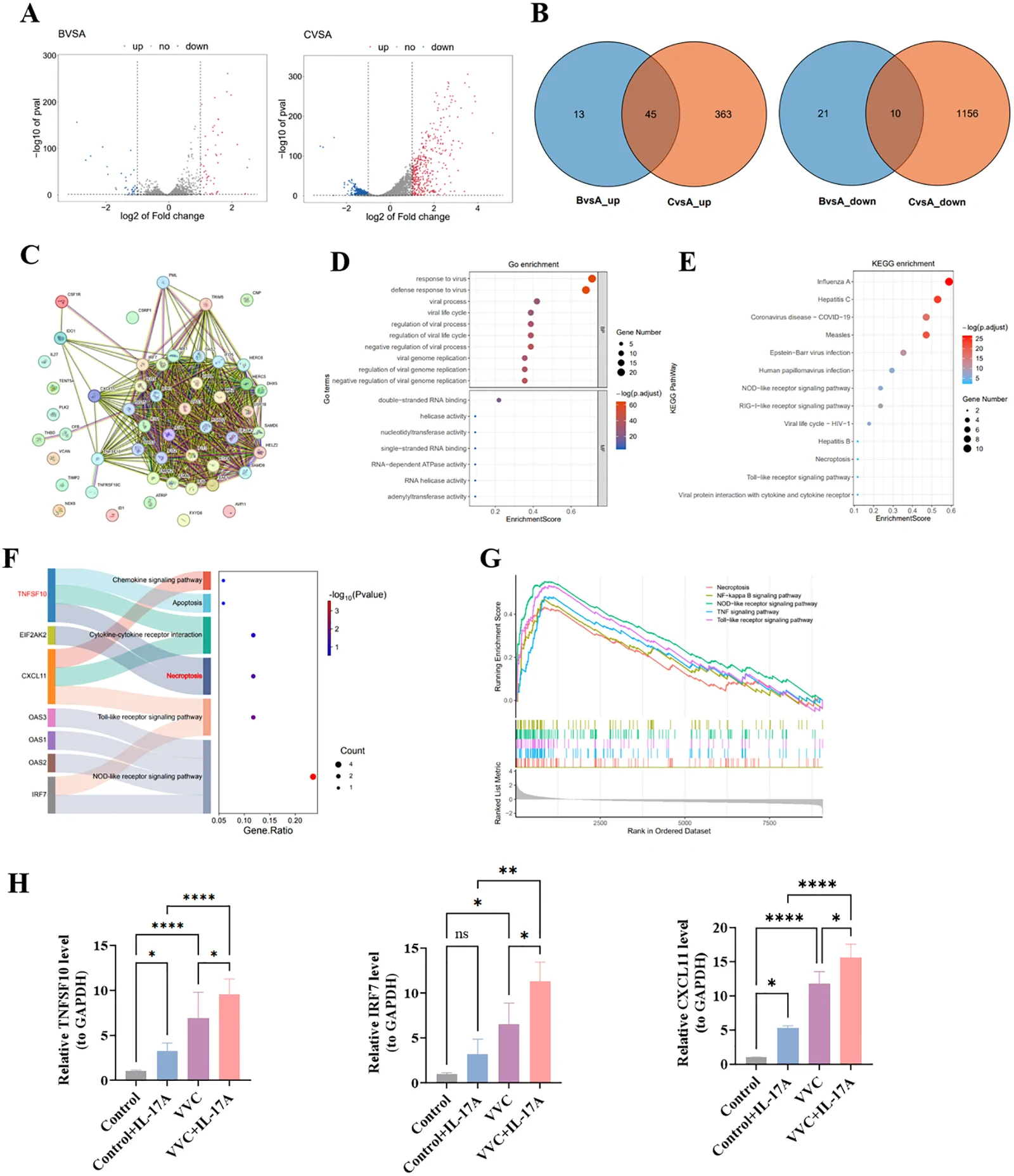
Transcriptomic analysis of vaginal neutrophils from healthy controls and patients with VVC. **(A)** Volcano plots of differentially expressed genes. **(B)** Venn analysis of shared DEGs. **(C)** Protein-protein interaction network. **(D)** Gene Ontology enrichment analysis. **(E)** KEGG pathway enrichment analysis. **(F)**Gene-pathway association analysis. **(G)** Gene-set enrichment analysis centered on *TNFSF10*. **(H)** qRT-PCR validation of TNFSF10, *IRF7*, and *CXCL11* transcript levels. *p < 0.05; **p < 0.01; ***p < 0.001; ****p < 0.0001.

Because IL-17A regulates neutrophil death in VVC (Shao et al., 2023) and TNFSF10 has been linked to both apoptosis and necroptosis (Simoes Eugénio et al., 2021; Xue et al., 2025), TNFSF10 was selected for subsequent mechanistic evaluation.

### 3.3 IL-17A activates necroptosis signaling in neutrophils

To evaluate the effect of IL-17A at the protein level, neutrophils isolated from vaginal lavage fluid of healthy donors were treated with IL-17A. Compared with untreated controls, IL-17A increased TNFSF10 and TNFRSF10 abundance and enhanced RIP3 and MLKL phosphorylation (**Figures 3A, B**). These molecular changes indicate activation of TNFSF10/TNFRSF10 and RIP3/MLKL-dependent necroptosis signaling.

**Figure 3:**
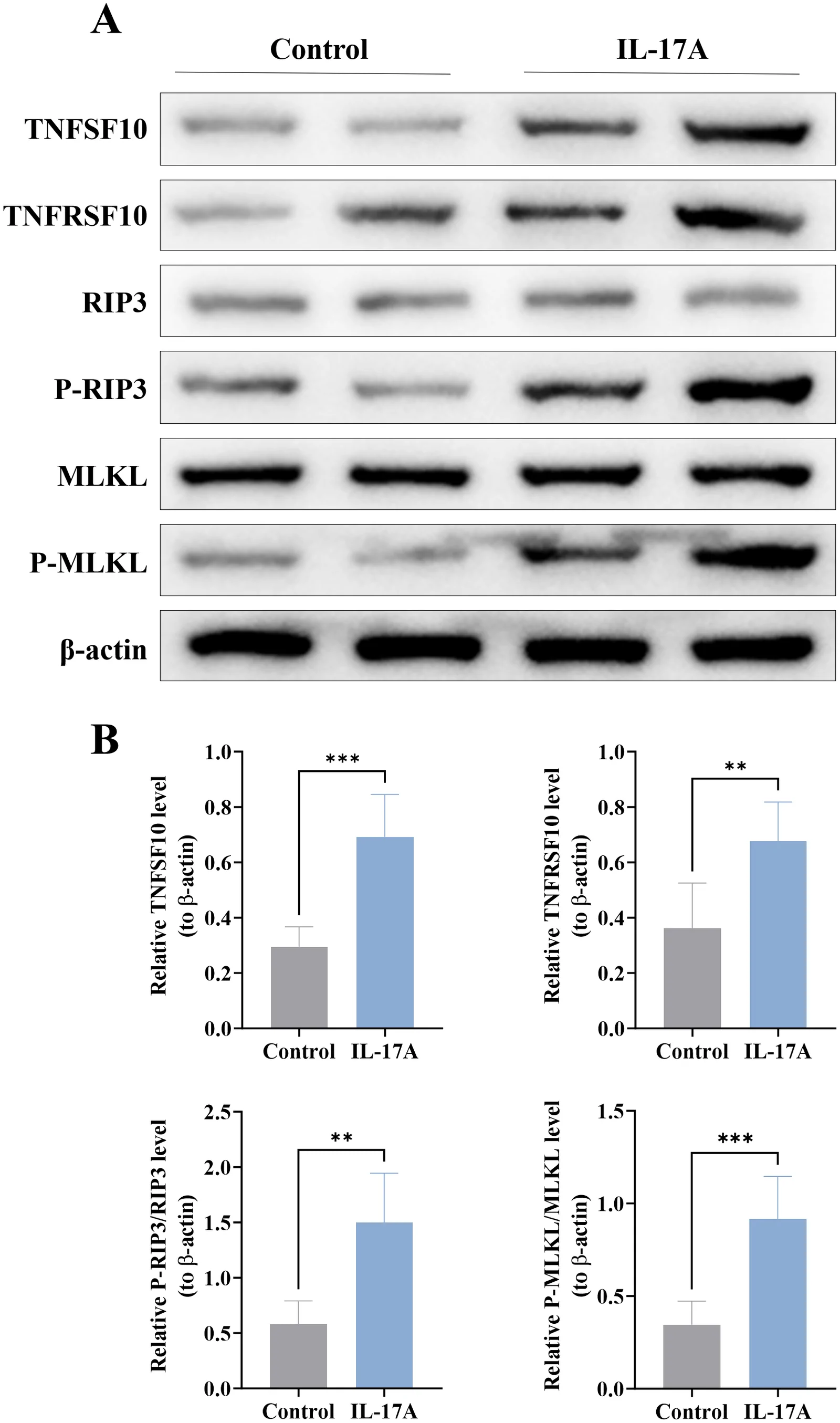
IL-17A activates TNFSF10/TNFRSF10 and necroptosis signaling in human vaginal neutrophils. **(A)** Western blot detection of TNFSF10, TNFRSF10, RIP3, p-RIP3, MLKL, and p-MLKL; β-actin served as the loading control. **(B)** Densitometric quantification relative to β-actin. *p < 0.05; **p < 0.01; ***p < 0.001; ****p < 0.0001.

### 3.4 Effects of TNFSF10 and IL-17A on the mucosal barrier and neutrophil infiltration in VVC

A mouse model of VVC was used to evaluate the effects of TNFSF10 and IL-17A on epithelial injury and neutrophil infiltration. Histological examination showed vaginal epithelial injury (**Figure 4D**), and ZO-1 immunohistochemistry was used to assess barrier integrity (**Figure 4E**). ZO-1 expression was lower in VVC mice than in controls. TNFSF10 overexpression increased ZO-1, whereas TNFSF10 knockdown further reduced it. Exogenous IL-17A decreased ZO-1, while IL-17A neutralization partially restored expression. MPO, dsDNA, and TNFSF10 concentrations in vaginal lavage fluid were altered by VVC and by the respective interventions (**Figure 4F**). Flow cytometry confirmed increased neutrophil infiltration in VVC mice; TNFSF10 overexpression reduced neutrophil accumulation, TNFSF10 knockdown increased it, IL-17A further increased it, and IL-17A neutralization reduced it (**Figure 4G**). These independent *in vivo* findings were complemented by the four-group cell-based rescue analysis integrated into **Figure 5**.

**Figure 4:**
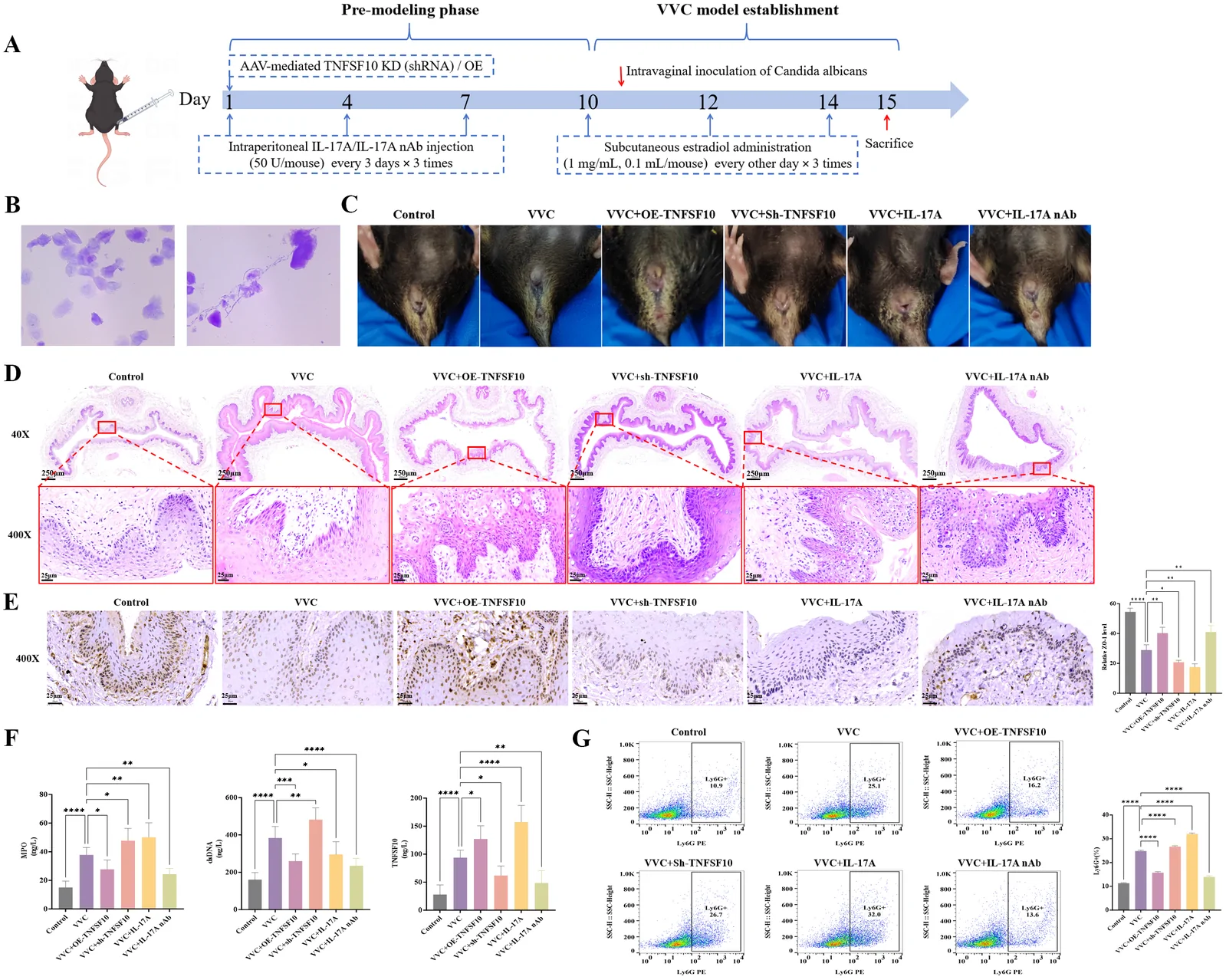
Independent effects of TNFSF10 and IL-17A interventions in a mouse model of VVC. **(A)** Experimental timeline. **(B)** Vaginal lavage microscopy showing absence or presence of fungal hyphae. **(C)** External genital findings. **(D)** Hematoxylin and eosin staining at 40× and 400× magnification; scale bars = 250 mm and 25 mm, respectively. **(E)** ZO-1 immunohistochemistry at 400× magnification; scale bar = 25 mm. **(F)** Vaginal-lavage concentrations of MPO, dsDNA, and TNFSF10. **(G)** Flow-cytometric quantification of Ly6G+ neutrophils. *p < 0.05; **p < 0.01; ***p < 0.001; ****P < 0.0001.

**Figure 5:**
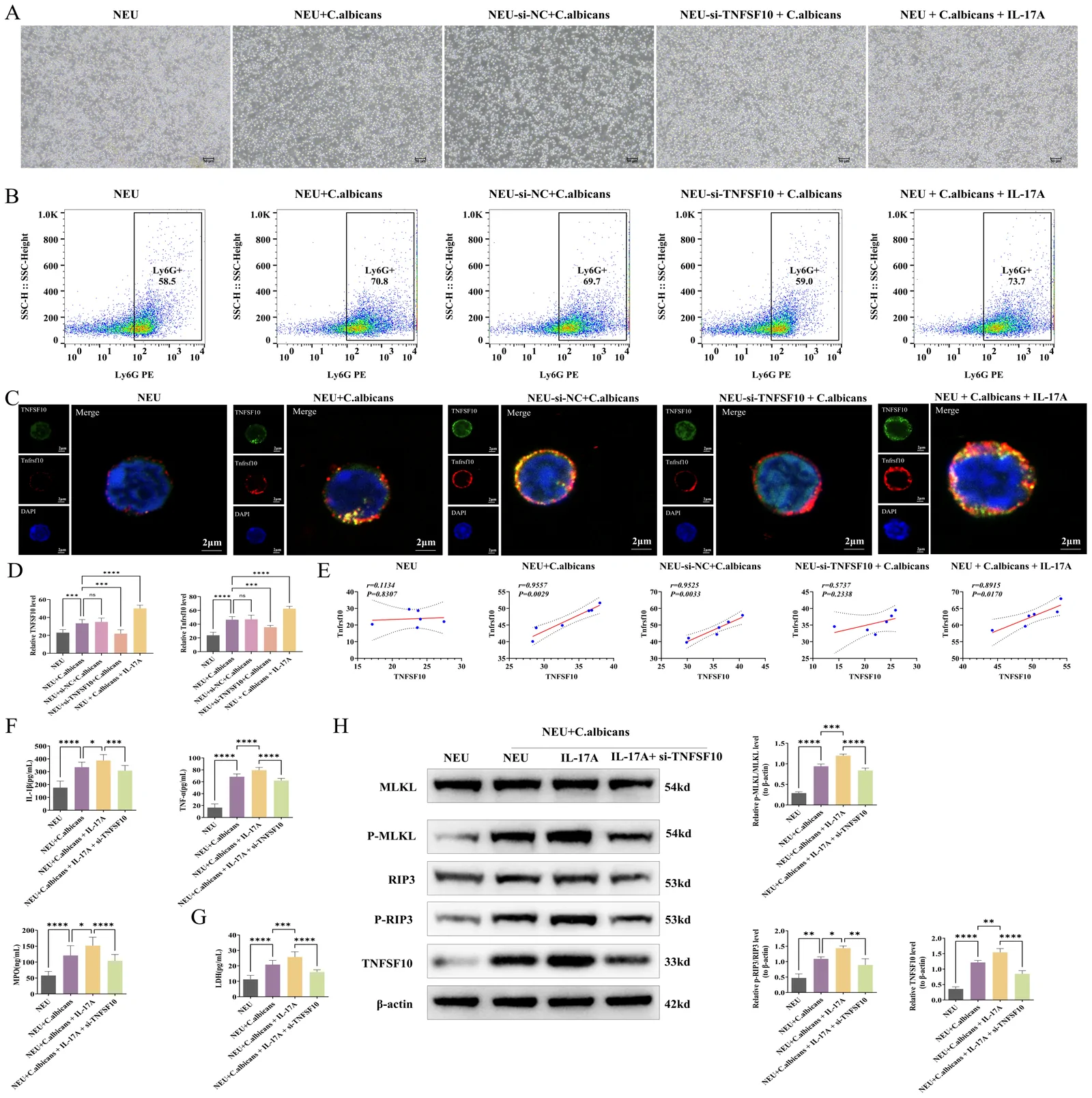
IL-17A enhances TNFSF10/TNFRSF10 signaling, whereas TNFSF10 knockdown attenuates IL-17A-induced inflammation and necroptosis during *C.albicans* exposure. **(A)** Morphology of isolated neutrophils; scale bar = 50 mm. **(B)** Flow-cytometric quantification of Ly6G^+^cells. **(C)** TNFSF10 (green), TNFRSF10 (red), and nuclear (blue) immunofluorescence; scale bar = 2 mm. **(D)** Quantification of TNFSF10 and TNFRSF10 fluorescence intensity. **(E)** Correlation between TNFSF10 and TNFRSF10 fluorescence intensity. The rescue panels compare Control, *C.albicans*, *C.albicans* + IL-17A, and *C.albicans* + IL-17A+si-TNFSF10 groups: **(F)** ELISA measurements of IL-1b and MPO in culture supernatants; **(G)** LDH release; and **(H)** Western blot analysis of TNFSF10, RIP3, p-RIP3, MLKL, and p-MLKL, with b-actin as the loading control. Data are presented as mean ± SEM. ns, not significant; *p < 0.05; **p < 0.01; ***p < 0.001; ****p < 0.0001.

### 3.5 Effects of IL-17A and TNFSF10 on neutrophil activation during *C. albicans* infection

To examine the direct effects of IL-17A and TNFSF10 during fungal exposure, mouse peripheral-blood neutrophils were assigned to five groups: untreated control, *C. albicans* co-culture, negative-control siRNA plus *C. albicans*, TNFSF10 siRNA plus *C. albicans*, and *C. albicans* plus exogenous IL-17A. Ly6G^+^ cells constituted more than 87% of each preparation (**Figure 5B**). *C. albicans* increased TNFSF10 and TNFRSF10 immunofluorescence and their colocalization; TNFSF10 knockdown reduced both signals, whereas IL-17A further increased them (**Figures 5C, D**). TNFSF10 and TNFRSF10 fluorescence intensities were positively correlated in the fungal co-culture and IL-17A-treated groups (**Figure 5E**). These results demonstrate that IL-17A amplifies TNFSF10/TNFRSF10 signaling during fungal exposure.

Four-group rescue analysis further showed that *C. albicans* increased IL-1β and MPO concentrations, LDH release, TNFSF10 expression, and phosphorylation of RIP3 and MLKL; IL-17A further enhanced these responses. Addition of si-TNFSF10 markedly attenuated the IL-17A-induced increases in IL-1β, MPO, and LDH release and reduced TNFSF10 expression and the p-RIP3/RIP3 and p-MLKL/MLKL ratios (**Figures 5F–H**). These findings support TNFSF10 as a downstream mediator of IL-17A-induced neutrophil inflammation, membrane injury, and necroptosis during *C. albicans* exposure.

### 3.6 Effects of TNFSF10 and IL-17A on neutrophil function and death during *C. albicans* exposure

*C. albicans* co-culture increased neutrophil migration (**Figure 6A**), whereas TNFSF10 knockdown reduced migration and exogenous IL-17A further enhanced it. Concentrations of IL-1β, IL-17A, MPO, and TNF-α increased after fungal exposure and showed additional group-dependent changes after TNFSF10 knockdown or IL-17A treatment (**Figure 6B**). qRT-PCR showed increased *Tnfsf10*, *Tnfrsf10*, *Ripk3*, and *Mlkl* transcripts after *C. albicans* exposure. TNFSF10 knockdown reduced these transcript changes, while IL-17A enhanced them (**Figure 6C**). Western blotting showed concordant changes in TNFSF10, TNFRSF10, RIP3, and MLKL abundance and in the p-RIP3/RIP3 and p-MLKL/MLKL ratios (**Figure 6D**). Together, these findings link TNFSF10 and IL-17A to neutrophil migration, inflammatory responses, and necroptosis during fungal exposure.

**Figure 6:**
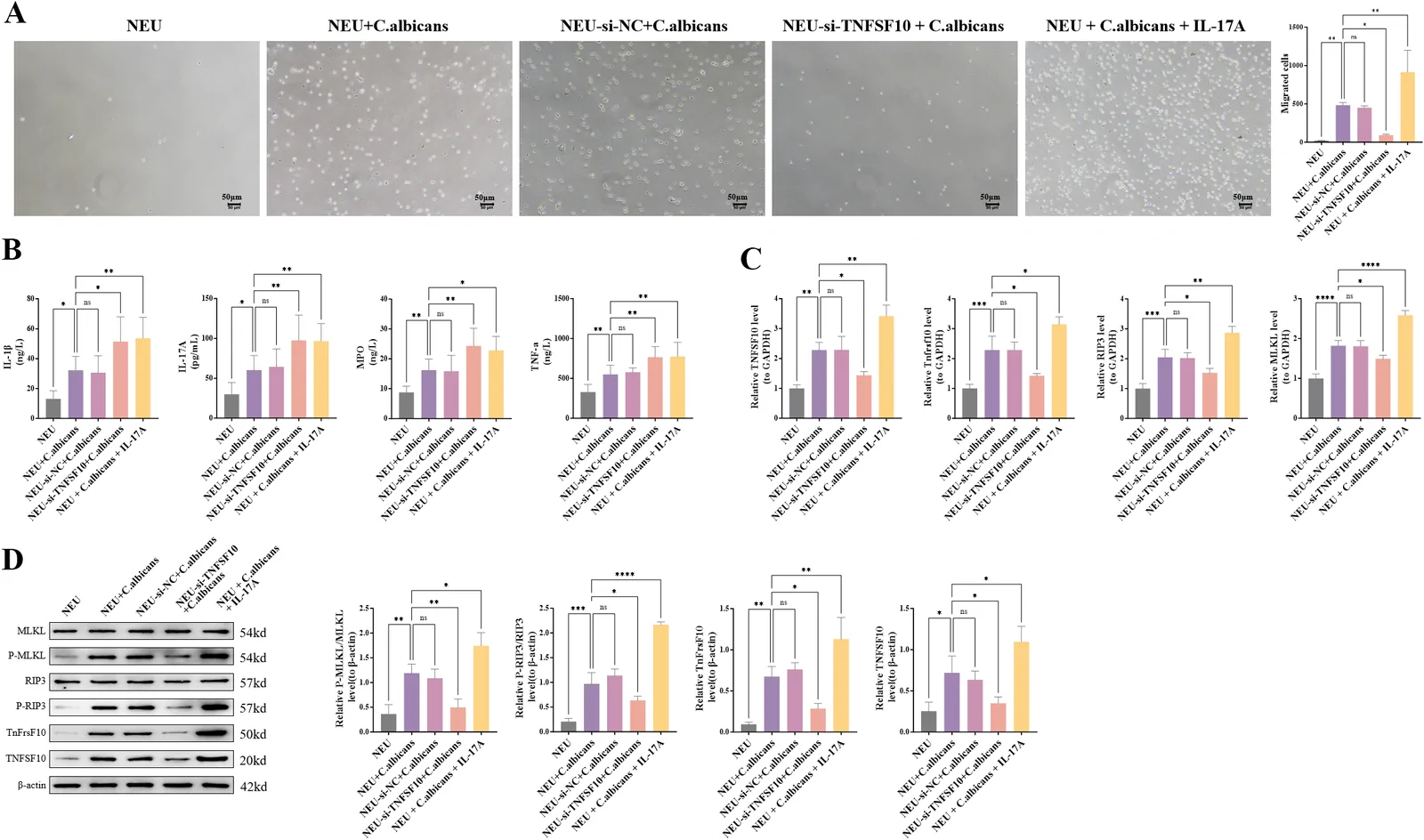
TNFSF10/TNFRSF10 signaling regulates neutrophil migration, inflammatory responses, and necroptosis. **(A)** Transwell migration assay; scale bar = 50 μm. **(B)** IL-1β, IL-17A, MPO, and TNF-α concentrations in culture supernatants. **(C)** Relative *Tnfsf10*, *Tnfrsf10*, *Ripk3*, and *Mlkl* transcript levels measured by qRT-PCR. **(D)** Western blot analysis of TNFSF10, TNFRSF10, RIP3, p-RIP3, MLKL, and p-MLKL, with β-actin as the loading control. Data are presented as mean ± SEM. ns, not significant; *p < 0.05; **p < 0.01; ***p < 0.001; ****p < 0.0001.

## 4 Discussion

The pathogenesis of VVC involves a complex imbalance in local mucosal immunity (Czechowicz et al., 2022). Neutrophils are central effector cells in vaginal host defense, but dysregulated recruitment, activation, and death can contribute to tissue injury (Roselletti et al., 2023; Costa-Fujishima et al., 2023). This study examined how IL-17A and TNFSF10 regulate NET formation, neutrophil activation, and necroptosis in clinical specimens, cell-based systems, and a mouse model, and further tested pathway order using TNFSF10-knockdown rescue experiments.

Previous studies showed that *C. albicans* infection induces IL-17A production in the vaginal mucosa and shapes neutrophil recruitment and activation. Consistent with the report by Zambrano et al. (2022), basal NET formation was higher in neutrophils from patients with VVC than in those from healthy controls. Exogenous IL-17A further increased NET formation. Transcriptomic screening followed by qRT-PCR and Western blot validation identified TNFSF10/TNFRSF10 and RIP3/MLKL signaling as a prominent neutrophil response to IL-17A. TNFSF10 can signal through death receptors and, in specific inflammatory contexts, participate in RIPK1/RIP3/MLKL- dependent necroptosis (Simoes Eugénio et al., 2021; Xue et al., 2025).

Vaginal epithelial barrier damage is a central pathological feature of VVC (Hasselrot et al., 2024), and ZO-1 expression reflects tight-junction integrity (Illek et al., 2023). ZO-1 was reduced in VVC mice, further decreased after TNFSF10 knockdown or IL-17A treatment, and increased after TNFSF10 overexpression or IL-17A neutralization. Although TNFSF10 can participate in inflammatory cell-death pathways (Simoes Eugénio et al., 2021; Chen et al., 2022; Berg et al., 2023), TNFSF10 overexpression in this model was accompanied by reduced neutrophil accumulation and improved ZO-1 expression. This pattern suggests that the net tissue effect of TNFSF10 depends on the balance between neutrophil recruitment, activation, and programmed cell death.

MPO and dsDNA measurements provided additional evidence of altered neutrophil activation and NET-associated processes (Rizo-Téllez et al., 2022; Burn et al., 2025). The reduction in dsDNA after exogenous IL-17A in the mouse model contrasted with the increase in NET formation observed in human clinical neutrophils. This discrepancy may reflect differences in species, disease stage, experimental compartment, or extracellular DNA clearance (Sun et al., 2023). It also indicates that IL-17A-dependent regulation of NET-associated readouts is context dependent and should not be interpreted from a single marker alone.

The four-group rescue experiment integrated into **Figure 5** strengthened the proposed pathway relationship. During *C. albicans* exposure, IL-17A increased ELISA-measured IL-1β and MPO, LDH release, TNFSF10 protein expression, and phosphorylation of RIP3 and MLKL. TNFSF10 silencing attenuated these changes. Together, the genetic rescue, inflammatory readouts, and LDH data support TNFSF10 as a downstream mediator of IL-17A-induced neutrophil inflammation, membrane injury, and necroptosis.

Several limitations should be acknowledged. First, no additional animal experiment was performed to test combined *in vivo* IL-17A/TNFSF10 intervention groups, such as VVC + IL-17A + sh-TNFSF10 or VVC + IL-17A + OE-TNFSF10. Therefore, although the four-group cell-based rescue experiment supports pathway order, *in vivo* dependence remains to be confirmed. Second, IL-17A transcript or intracellular protein abundance was not measured in purified vaginal neutrophils from healthy donors and patients with VVC; the present study instead used IL-17A as an exogenous upstream stimulus and evaluated downstream responses. Third, although genetic TNFSF10 intervention, RIP3/MLKL phosphorylation, and LDH release support neutrophil necroptosis, LDH is not specific to necroptosis and pharmacological rescue experiments using Necrostatin-1 or necrosulfonamide were not performed. Future studies should incorporate these inhibitors, combined *in vivo* rescue designs, and additional cell-death assays.

## 5 Conclusions

In conclusion, IL-17A is an important regulator of neutrophil activation in VVC. Across the experimental systems used here, IL-17A increased TNFSF10/TNFRSF10 expression and RIP3/MLKL phosphorylation and was associated with altered NET formation, neutrophil infiltration, and epithelial barrier injury. TNFSF10 knockdown attenuated IL-17A-induced necroptosis signaling, inflammatory mediator production, and LDH release in the rescue experiment, supporting TNFSF10 as a downstream effector of IL-17A. These findings identify the IL-17A/TNFSF10 axis as a potential therapeutic target in VVC, while inhibitor-based studies are required for further confirmation.

## Data Availability

The datasets presented in this study can be found in online repositories. The names of the repository/repositories and accession number(s) can be found in the article/**Supplementary Material**.
