# Supplementary material for: The IL-17A/TNFSF10 axis promotes vulvovaginal candidiasis pathogenesis by inducing neutrophil necroptosis
Source: Front Cell Infect Microbiol. 2026 Sep 24;16:1910964. doi: 10.3389/fcimb.2026.1910964 (PMC13645517; doi:10.3389/fcimb.2026.1910964)
Supplement: Supplementary file 1 [file DataSheet1.pdf]

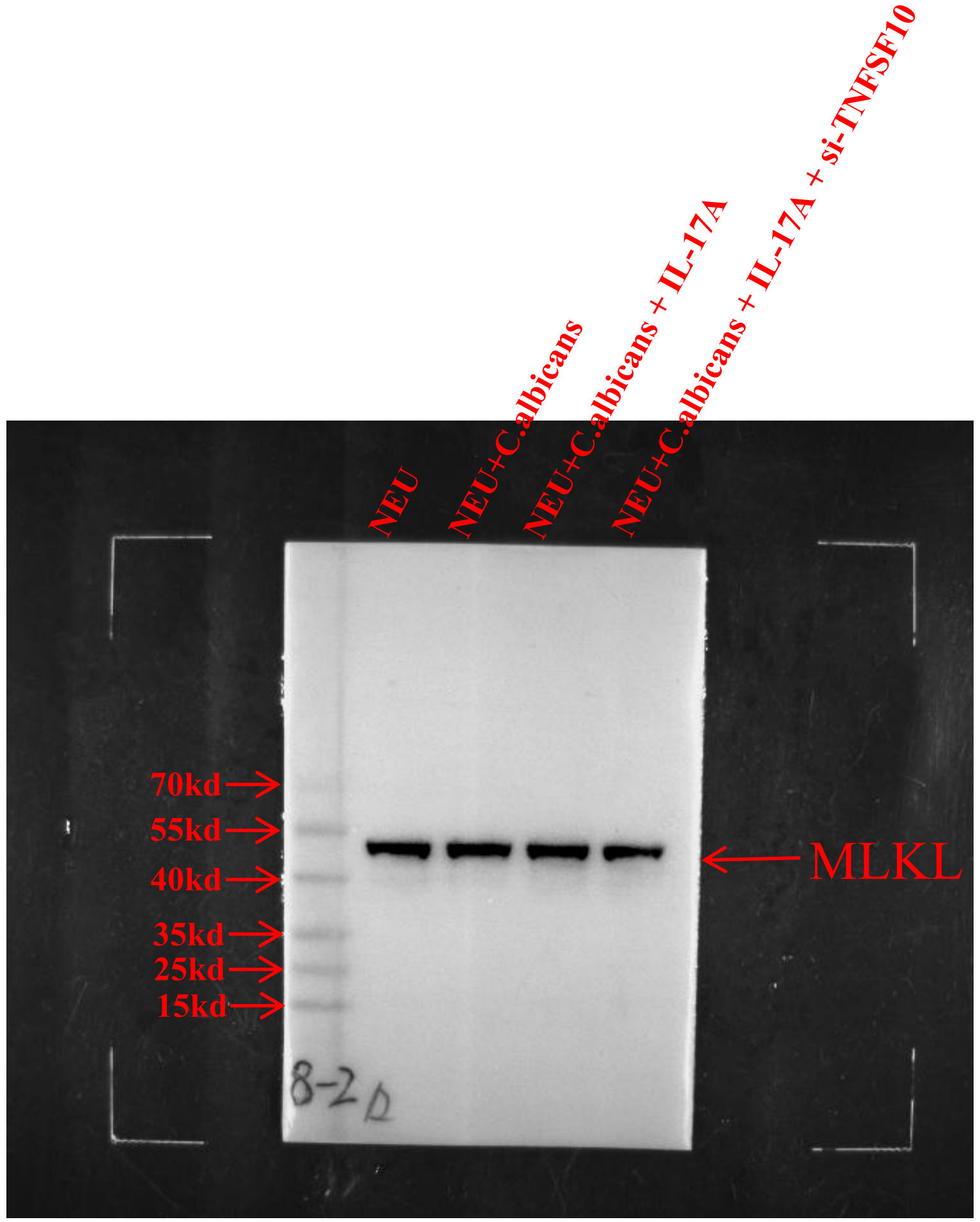

NEU
NEU+C.albicans
NEU+C.albicans + IL-17A
NEU+C.albicans + IL-17A + si-TNFSF10
70kd
55kd
40kd
35kd
25kd
15kd
MLKL
8-2

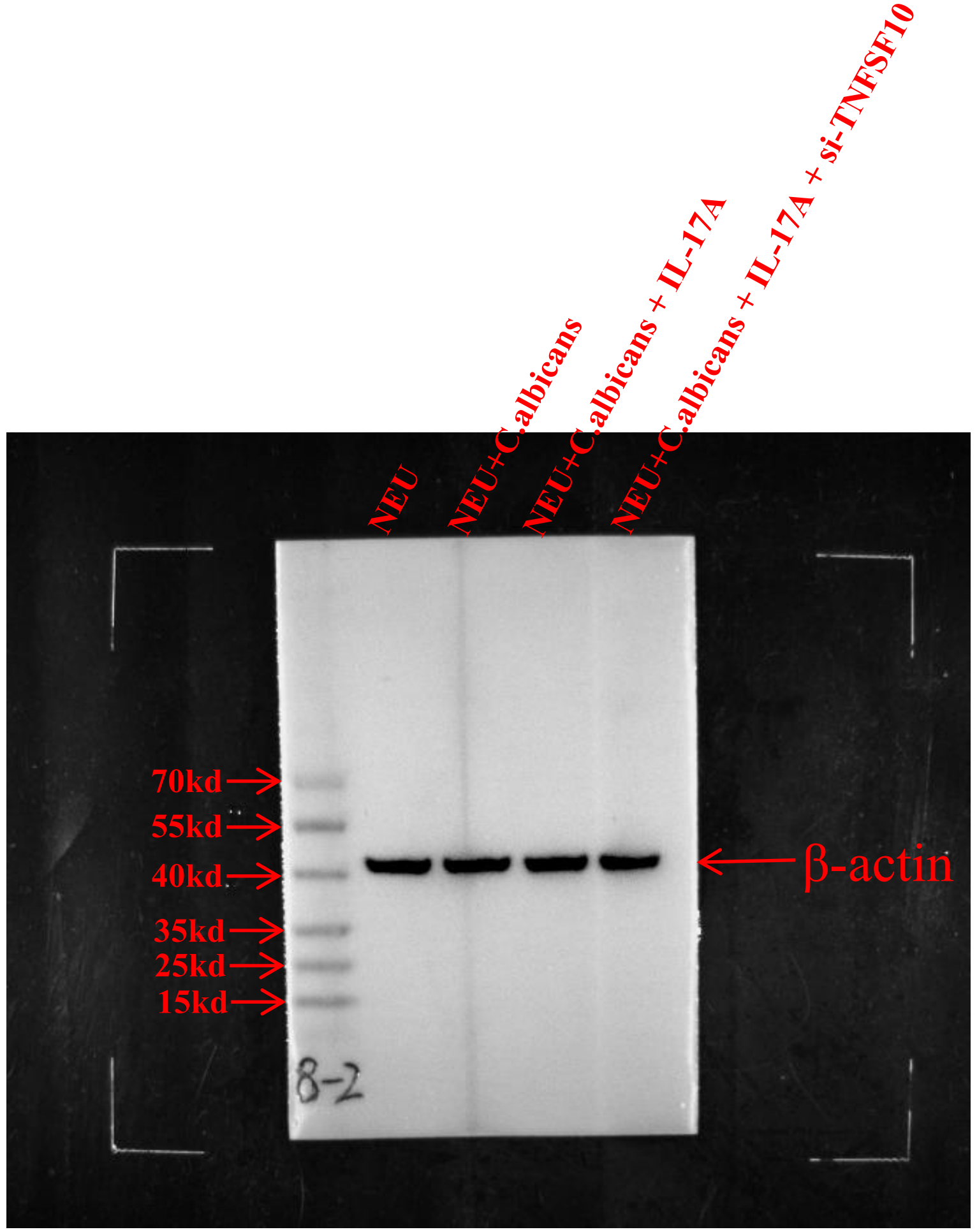

NEU
NEU+C.albicans
NEU+C.albicans + IL-17A
NEU+C.albicans + IL-17A + si-TNFSF10
70kd
55kd
40kd
35kd
25kd
15kd
β-actin
8-2

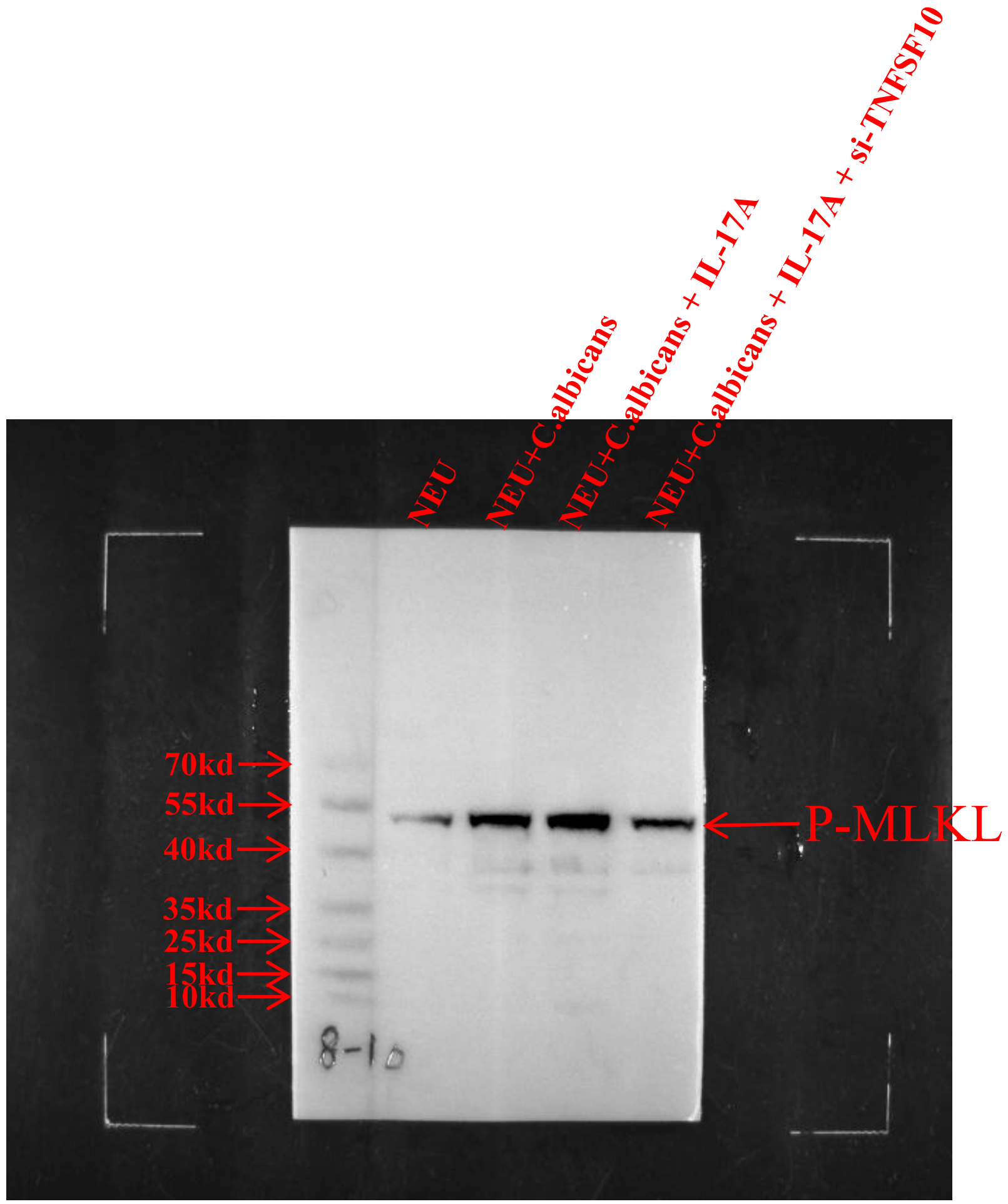
NEU
NEU+C.albicans
NEU+C.albicans + IL-17A
NEU+C.albicans + IL-17A + si-TNFSF10
70kd
55kd
40kd
35kd
25kd
15kd
10kd
P-MLKL
8-10

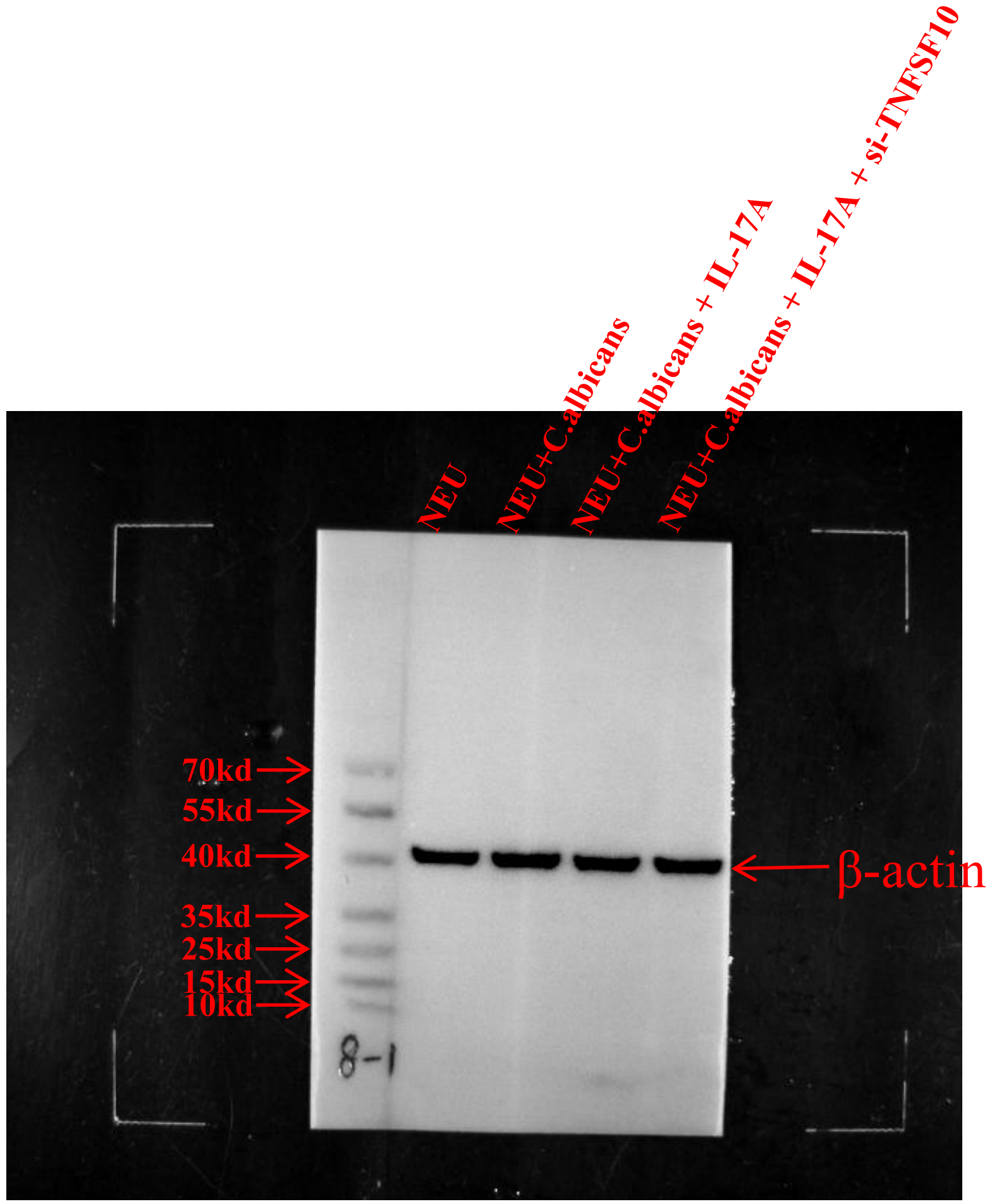
NEU
NEU+C.albicans
NEU+C.albicans + IL-17A
NEU+C.albicans + IL-17A + si-TNFSF10
70kd
55kd
40kd
35kd
25kd
15kd
10kd
β-actin
8-1

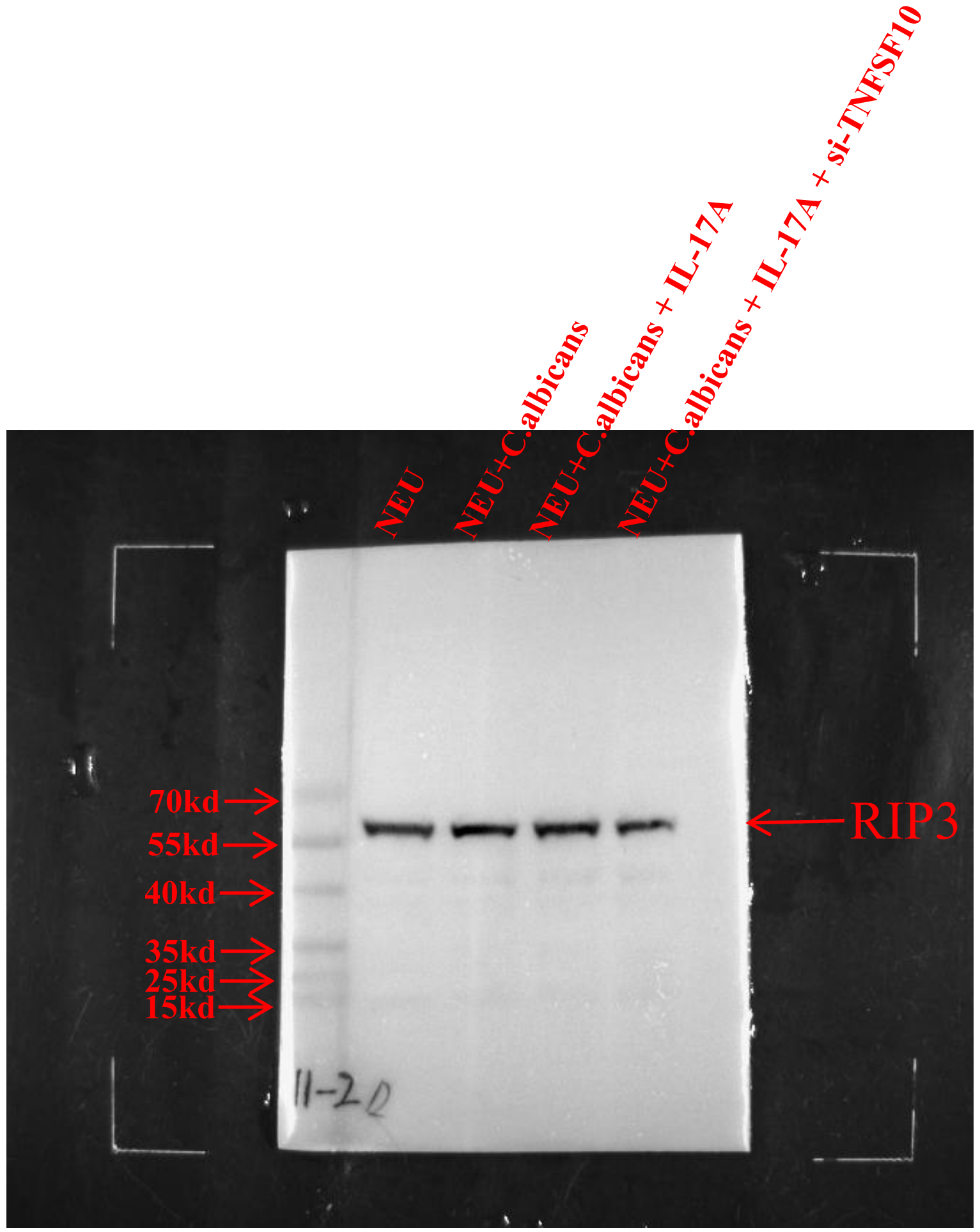
NEU
NEU+C.albicans
NEU+C.albicans + IL-17A
NEU+C.albicans + IL-17A + si-TNFSF10
70kd
55kd
40kd
35kd
25kd
15kd
RIP3
11-2

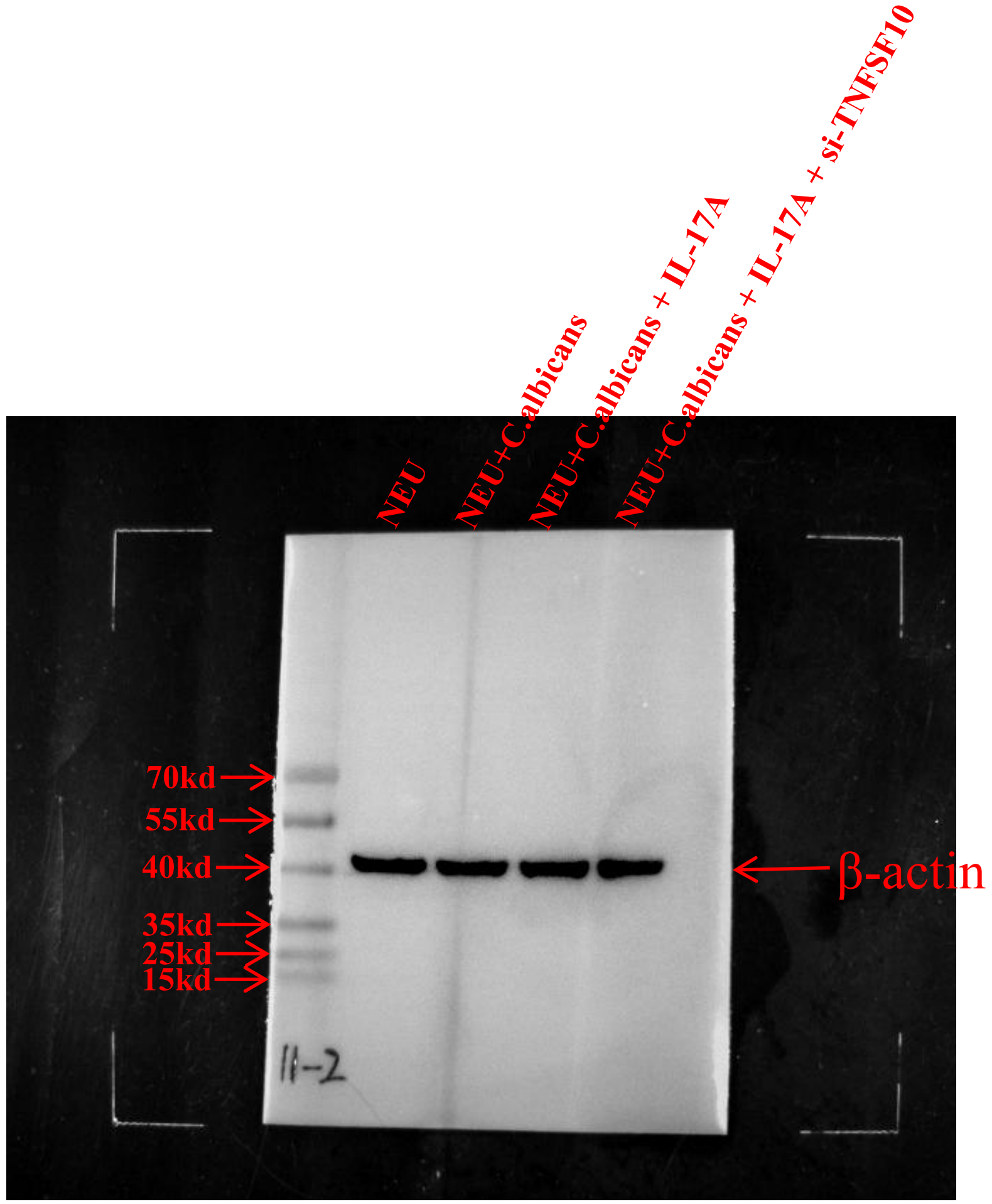
NEU
NEU+C.albicans
NEU+C.albicans + IL-17A
NEU+C.albicans + IL-17A + si-TNFSF10
70kd
55kd
40kd
35kd
25kd
15kd
β-actin
11-2

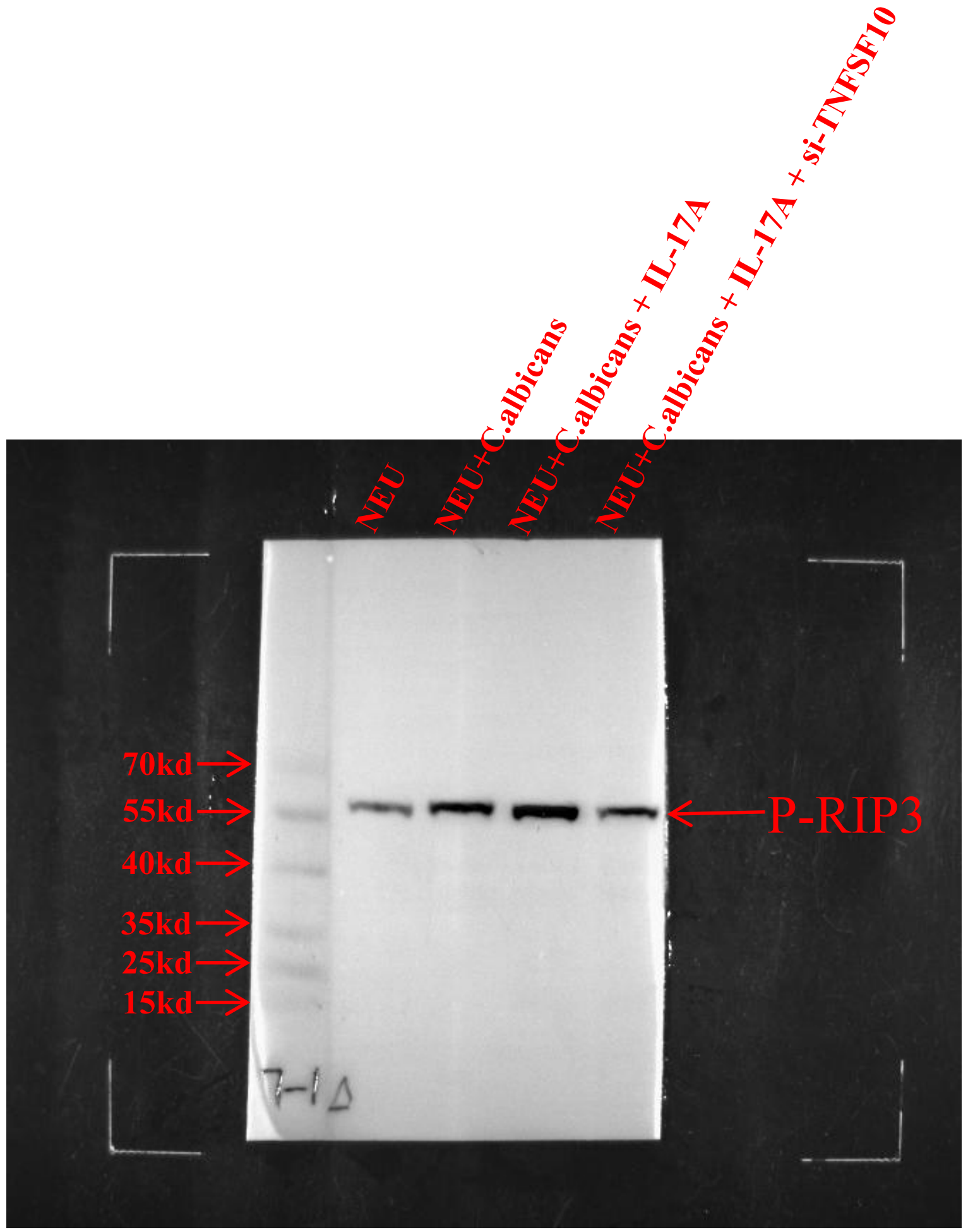
NEU
NEU+C.albicans
NEU+C.albicans + IL-17A
NEU+C.albicans + IL-17A + si-TNFSF10
70kd
55kd
40kd
35kd
25kd
15kd
P-RIP3
7-1

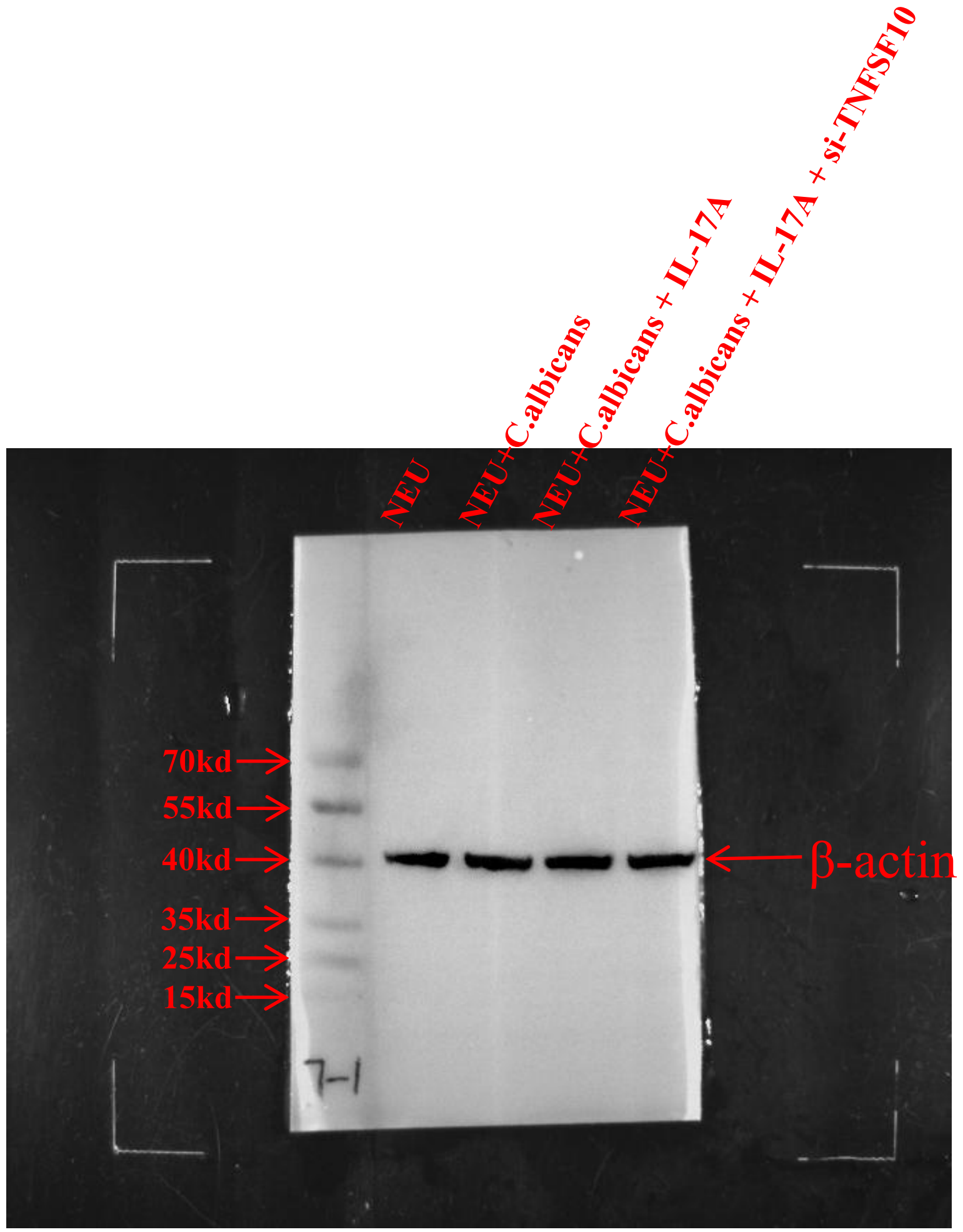
NEU
NEU+C.albicans
NEU+C.albicans + IL-17A
NEU+C.albicans + IL-17A + si-TNFSF10
70kd
55kd
40kd
35kd
25kd
15kd
β-actin
7-1

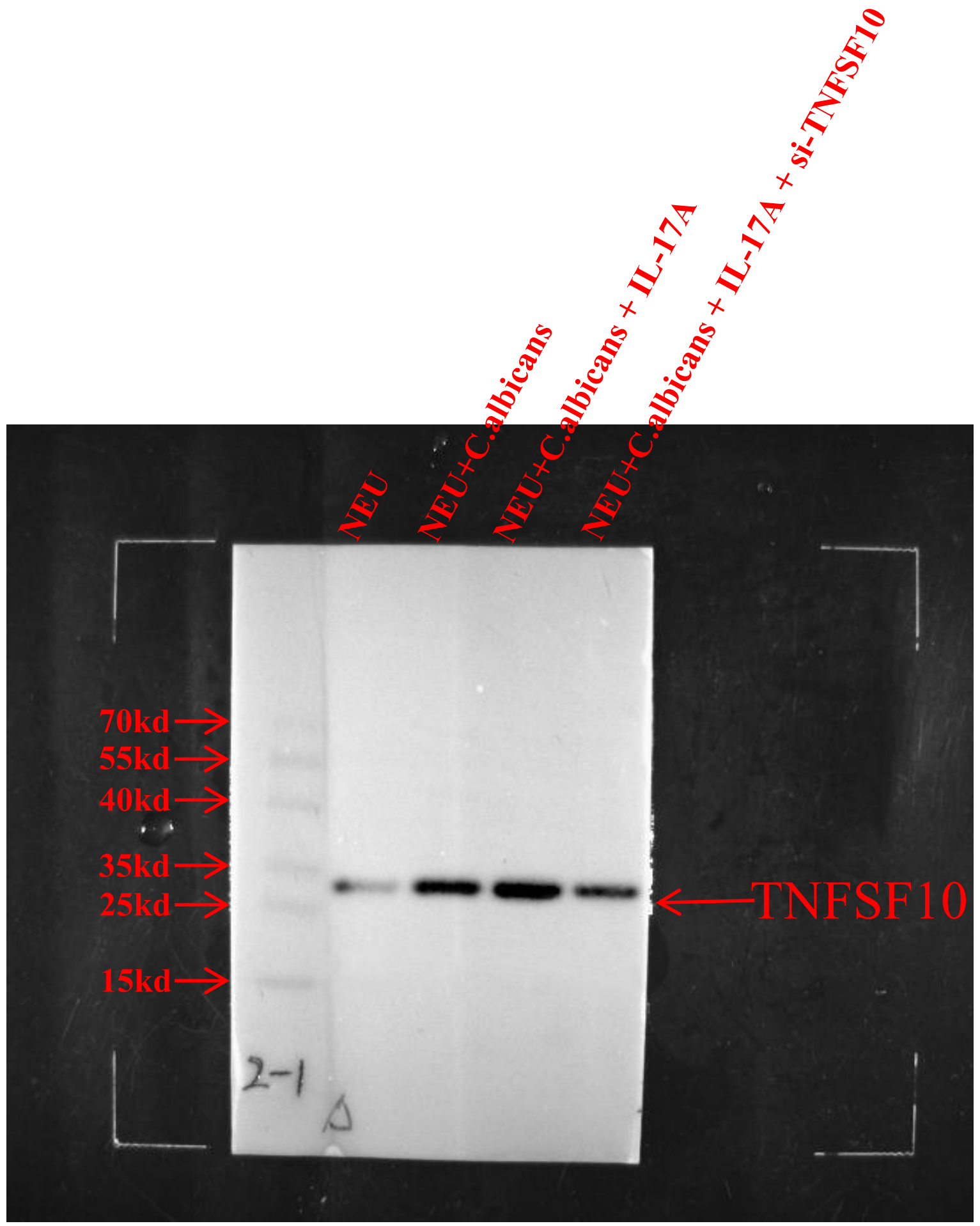
NEU
NEU+C.albicans
NEU+C.albicans + IL-17A
NEU+C.albicans + IL-17A + si-TNFSF10
70kd
55kd
40kd
35kd
25kd
15kd
TNFSF10
2-1

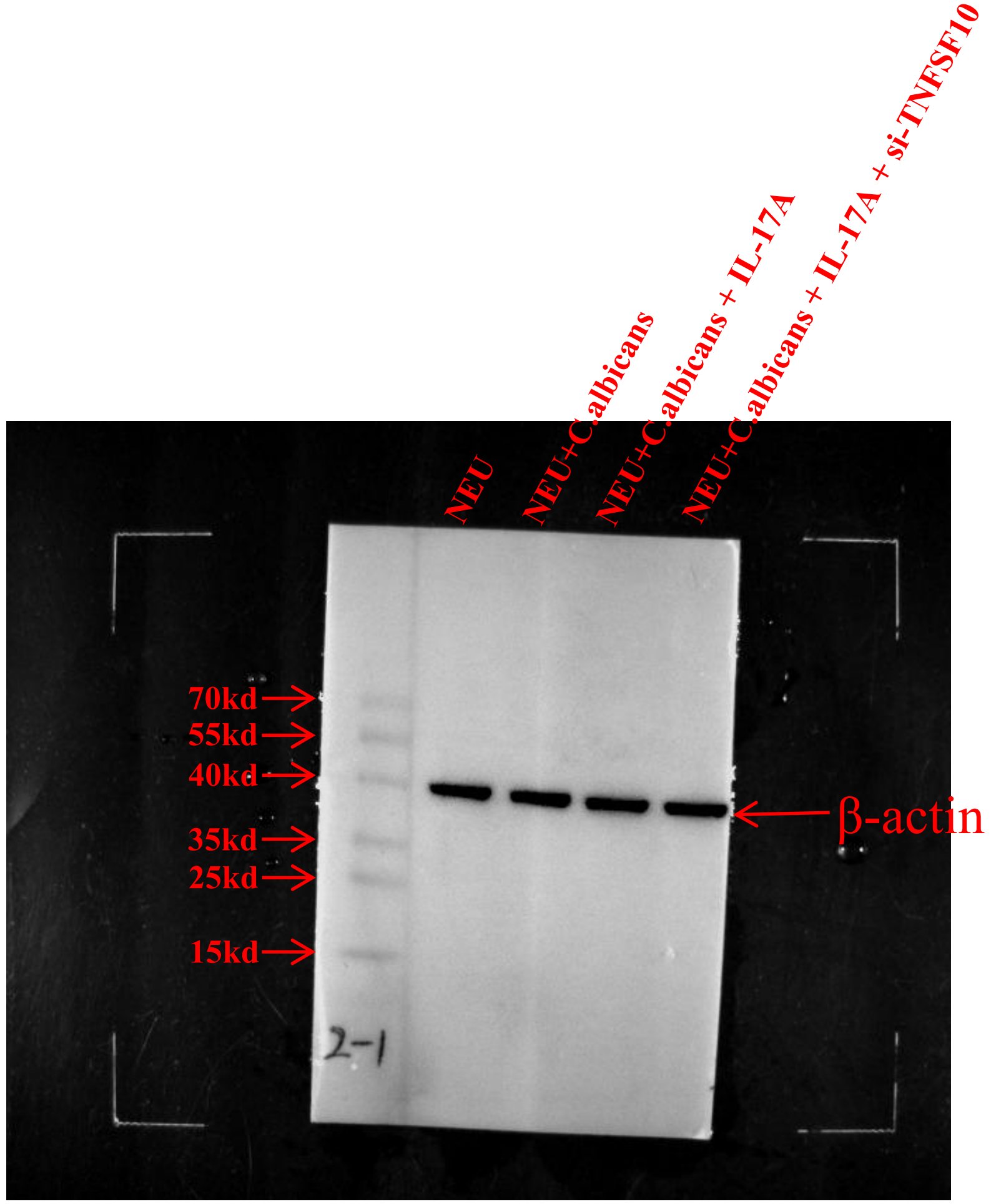
NEU
NEU+C.albicans
NEU+C.albicans + IL-17A
NEU+C.albicans + IL-17A + si-TNFSF10
70kd
55kd
40kd
35kd
25kd
15kd
β-actin
2-1
